# Exon-focused genome-wide association study of obsessive-compulsive disorder and shared polygenic risk with schizophrenia

**DOI:** 10.1038/tp.2016.34

**Published:** 2016-03-29

**Authors:** J Costas, N Carrera, P Alonso, X Gurriarán, C Segalàs, E Real, C López-Solà, S Mas, P Gassó, L Domènech, M Morell, I Quintela, L Lázaro, J M Menchón, X Estivill, Á Carracedo

**Affiliations:** 1 Grupo de Xenética Psiquiátrica, Instituto de Investigación Sanitaria de Santiago, Complexo Hospitalario Universitario de Santiago de Compostela, Servizo Galego de Saúde, Santiago de Compostela, Spain; 2 Centre for Neuropsychiatric Genetics and Genomics, Institute of Psychological Medicine and Clinical Neurosciences, Cardiff University, Cardiff, UK; 3 Fundación Pública Galega de Medicina Xenómica, Servicio Galego de Saúde, Instituto de Investigación Sanitaria de Santiago de Compostela, Santiago de Compostela, Spain; 4 OCD Clinical and Research Unit, Department of Psychiatry, Hospital de Bellvitge, Barcelona, Spain; 5 Bellvitge Biomedical Research Institute-IDIBELL, Barcelona, Spain; 6 Centro de Investigación Biomédica en Red de Salud Mental, Carlos III Health Institute, Madrid, Spain; 7 Department of Clinical Sciences, Bellvitge Campus, University of Barcelona, Barcelona, Spain; 8 Grupo de Medicina Xenómica, Universidade de Santiago de Compostela, Santiago de Compostela, Spain; 9 Department of Anatomic Pathology, Pharmacology and Microbiology, University of Barcelona, Barcelona, Spain; 10 Institut d'Investigacions Biomèdiques August Pi i Sunyer, Barcelona, Spain; 11 Department of Psychiatry and Clinical Psychobiology, University of Barcelona, Barcelona, Spain; 12 Genomics and Disease Group, Centre for Genomic Regulation, Barcelona, Spain; 13 Department of Experimental and Health Sciences, Universitat Pompeu Fabra, Barcelona, Spain; 14 Centro de Investigación Biomédica en Red Epidemiología y Salud Pública, Centre for Genomic Regulation, Barcelona, Spain; 15 Hospital del Mar Research Institute (IMIM), Barcelona, Spain; 16 Grupo de Medicina Xenómica, Universidade de Santiago de Compostela, Centro Nacional de Genotipado - Instituto Carlos III, Santiago de Compostela, Spain; 17 Centro de Investigación Biomédica en Red de Enfermedades Raras, Santiago de Compostela, Spain; 18 Department of Child and Adolescent Psychiatry and Psychology, Institute of Neurosciences, Hospital Clínic Universitari, Barcelona, Spain

## Abstract

Common single-nucleotide polymorphisms (SNPs) account for a large proportion of the heritability of obsessive-compulsive disorder (OCD). Co-ocurrence of OCD and schizophrenia is commoner than expected based on their respective prevalences, complicating the clinical management of patients. This study addresses two main objectives: to identify particular genes associated with OCD by SNP-based and gene-based tests; and to test the existence of a polygenic risk shared with schizophrenia. The primary analysis was an exon-focused genome-wide association study of 370 OCD cases and 443 controls from Spain. A polygenic risk model based on the Psychiatric Genetics Consortium schizophrenia data set (PGC-SCZ2) was tested in our OCD data. A polygenic risk model based on our OCD data was tested on previous data of schizophrenia from our group. The most significant association at the gene-based test was found at *DNM3* (*P*=7.9 × 10^−5^), a gene involved in synaptic vesicle endocytosis. The polygenic risk model from PGC-SCZ2 data was strongly associated with disease status in our OCD sample, reaching its most significant value after removal of the major histocompatibility complex region (lowest *P*=2.3 × 10^−6^, explaining 3.7% of the variance). The shared polygenic risk was confirmed in our schizophrenia data. In conclusion, *DNM3* may be involved in risk to OCD. The shared polygenic risk between schizophrenia and OCD may be partially responsible for the frequent comorbidity of both disorders, explaining epidemiological data on cross-disorder risk. This common etiology may have clinical implications.

## Introduction

Obsessive-compulsive disorder (OCD) is a clinically heterogeneous neuropsychiatric disorder characterized by recurrent intrusive thoughts causing distress or anxiety, and compulsions, defined as repetitive behaviors or mental acts performed to alleviate this distress. The prevalence is around 1–3%, and the onset is bimodal during infancy or young adulthood.^1^ Twin studies revealed a considerable effect of genetic additive factors, with an estimated heritability of 42–52%.^2^ Co-occurrence of OCD and other related disorders that present overlapping or similar features and symptoms, such as Tourette's syndrome, is commoner than expected based on their respective prevalences, suggesting shared genetic susceptibility.^3^ A recent meta-analysis confirmed that there is also a larger prevalence rate of OCD in schizophrenia patients compared with the general population.^4^ Both the presence of a prior diagnosis of OCD as well as the presence of an OCD diagnosis in close relatives are associated with an increased risk of developing schizophrenia.^5, 6^ Atypical antipsychotics may induce obsessive-compulsive symptoms in susceptible schizophrenic patients.^7^ These findings suggest common etiological mechanisms that may have clinical implications, deserving further research.

A recent review of association studies at candidate genes in OCD has identified some evidence of association at *SLC6A4*, *HTR2A* and, in males only, *MAOA* and *COMT.*^8^ Linkage analysis has not revealed a causal locus. These results suggest that, as for other psychiatric disorders, OCD is a complex disorder that arises by the combination of many genetic and environmental factors of individual low effect. Two genome-wide association studies (GWASs) of OCD have been published to date.^9, 10^ Although the sample sizes were not large enough to detect significant single-nucleotide polymorphisms (SNPs) at the stringent genome-wide level, several interesting genes emerged for the follow-up studies. Additional analyses on these data have revealed that common SNPs account for a large proportion of the heritability and that a considerable proportion of the polygenic risk was shared between OCD and Tourette's syndrome.^11, 12^ In the present study, we describe an exon-based GWAS on OCD samples from Spain, and test the hypothesis that co-occurrence of OCD and schizophrenia may be partly owing to shared common SNPs of susceptibility.

## Materials and methods

### Samples

The case sample included 433 patients (227 males and 206 females) diagnosed with OCD. The onset was before the age of 15 years for 38% of the patients, and before 12 years of age for 21%. Diagnosis was done by two experienced psychiatrists following the SCID-CV (Structured Clinical Interview for DSM-IV Axis I Disorders-Clinician Version) in adults and the K.SADS-PL (The Schedule for affective Disorders and Schizophrenia for School-age Children-present and lifetime version) in pediatric group. The cases were recruited between 2003 and 2012 at the OCD Unit from the Bellvitge Hospital and the Department of Child and Adolescent Psychiatry and Psychology of Hospital Clínic, both from the Barcelona (Spain) health care area. All the patients are of European ancestry from Spain. The exclusion criteria were active psychoactive drugs dependence, psychotic disorders, intellectual disability and severe organic or neurologic pathology except tic disorders.

The control sample comprises 484 subjects (282 males and 202 females) from the National DNA Bank Carlos III (Salamanca University) and individuals attending primary health care centers in Galicia (NW Spain). They were all healthy unrelated individuals declaring not to suffer any disease and being subjected to a brief medical examination and a questionnaire. These controls are different from those of the Carrera *et al.* study^13^ used in polygenic risk analysis (see below). Informed consent was obtained for all the participants, and the research was done according to the principles of the Declaration of Helsinki after approval by the appropriate Ethic Committees (the Bellvitge University Hospital Ethical Committee, Barcelona, Spain; the Hospital Clínic Ethical Committee, Barcelona, Spain; and the Galician Ethical Committee for Clinical Research, Santiago de Compostela, Spain).

### Genotyping

The Axiom Exome Array (Affymetrix, Santa Clara, CA, USA) was used for genotyping cases and controls, following the manufacturer's instructions. The panel includes around 300 000 variants located in coding regions, GWAS tags from the NHGRI Catalog of Published Associations (August 2011), as well as ancestral informative markers. To minimize any batch effect, all the arrays contained approximately the same number of cases and controls. Variant call was performed by the Affymetrix Genotyping Console Software using the Axiom GT1 algorithm.

### Quality control

Extensive quality control filtering of the obtained genotypes was performed using standard procedures in PLINK.^14^ In particular, SNPs were removed from the study if they did not pass any of the following filters: (i) genotyping call rate above 95%, (ii) no significant departures from Hardy–Weinberg equilibrium in control samples (*P*>1 × 10^−3^); and (iii) no significant difference in call rate between cases and controls (*P*>1 × 10^−3^).

The samples presenting any of these conditions were removed: (i) call rate below 95% (ii) discordant gender between the one recorded in our database and the one inferred from the genotypes; (iii) heterozygosity levels departing three standard deviations from the mean; (iv) cryptic relatedness, detected by proportion identity-by-descent (PI_HAT) values greater than 0.05, as recommended by PLINK. In that case, the sample of the pair with lower call rate was removed. Finally, the genotype data of 3410 ancestral informative markers present in our samples were used to identify individuals with less than 90% Spanish ancestry using Structure v2.3.3 (ref. 15) and the HapMap samples from European ancestry (CEU), African ancestry (YRI) and Asian ancestry (JPT+CHB) as reference sets. The structure was run under the admixture model with 100 000 replications for burnin period and 100 000 replications after burnin for parameter estimations.

### Imputation

Imputation was performed for autosomal chromosomes following a pre-phasing/imputation stepwise approach with IMPUTE2/SHAPEIT, using default parameters.^16, 17^ As recommended, the chromosomes were divided in chunks of 5 Mb for the imputation and all the SNPs with minor allele frequency (MAF) over 0.01 were included as the input (94 096 variants). The 1000 Genome Project data set was used as the reference.^18^ After imputation, only the genotypes with an imputation info score >0.9 were considered for further analysis. Any SNP with imputation data on less than 95% of the sample was removed for further analysis.

### Association analysis at individual SNP level

After imputation, association analysis at SNP level was performed using logistic regression under an additive model, considering those SNPs at MAF>5%. The first 10 dimensions of multidimensional scaling, calculated from genotyped data at MAF>5%, were included as covariates to control for population stratification. The analysis was performed as implemented in PLINK 1.9. Manhattan plots and quantile-quantile plots were created with the R package qqman (https://github.com/stephenturner/qqman). Meta-analysis with previous GWAS data was performed using METAL.^19^

### Gene-based association analysis

VEGAS2 was used to perform gene-based tests from genotyped SNPs at MAF>0.1% in our samples using the exonic regions as gene boundaries.^20^ The SNP *P*-values were those based on logistic regression using the first 10 multidimensional scaling dimensions as covariates. The SNP *P*-values are converted to upper-tail *χ*^2^ statistics with one degree of freedom and summed to calculate the gene-based test statistic. To account for linkage disequilibrium among the SNPs, the null distribution of the test was estimated by simulation from the 1000 Genomes European samples. Only those SNPs with MAF>1% in the 1000 Genomes Project were considered for analysis. The analysis was performed using the web-based version of VEGAS2. A Bonferoni's correction based on the number of genes tested was used as a formal criteria for consideration of significance, although this is clearly conservative, taking into account that many genes overlap along the genome.

### Polygenic risk scores

Polygenic risk analysis was performed as previously described.^21, 22^ Basically, a polygenic risk model was constructed from GWAS data on a discovery sample. The model included the associated allele and its effect, measured as the logarithm of the OR, at each one of the SNP under a specific threshold of association *P*-values (*P*_threshold_). SNPs with alleles A/T or C/G were excluded to avoid strand ambiguity. Several different *P*_threshold_, from 0.01 to 1 (that is, inclusion of all the SNPs) were considered. Correlated SNPs due to linkage disequilibrium were pruned, using the clumping algorithm of PLINK, considering an *r*^2^=0.2 and a window size of 500 kb. The polygenic risk model was tested on a target sample, obtaining a polygenic risk score for each sample as the sum of the number of risk alleles carried by that sample weighted by its effect. The significance of the results, based on a Wald test for the coefficient of the score, was tested by comparison of two logistic regression models, one considering only the first 10 dimensions of multidimensional scaling to control for stratification, and another considering additionally the polygenic risk score. Nagelkerke's pseudo-*R*^2^ was calculated as a measure of the variance explained on the observed scaled.

Two different analyses were done, using PRSice.^23^ The first one considered the discovery phase of the second Psychiatric Genomics Consortium schizophrenia case–control mega-analysis, Psychiatric Genetics Consortium schizophrenia data set (PGC-SCZ2),^24^ as discovery sample and our OCD case–control samples as the target sample. By this way, the existence of common genetic susceptibility for both disorders was tested. Only genotyped SNPs or imputed SNPs with an imputation info score >0.9 were selected from the schizophrenia data. As an internal control, 100 permutations of the case–control labels at the OCD data were used as target samples.

The second analysis used the OCD data generated at this work as discovery sample and our previous data of a schizophrenia–control study on exonic SNPs using the Affymetrix 20k cSNPs array as target sample^13^ to test for the shared polygenic risk in an additional sample. The OCD data in polygenic risk analysis were restricted to all genotyped autosomal SNPs.

Power calculation for polygenic risk analysis was performed by the method of Palla and Dudbrigde (2015),^25^ as implemented in AVENGEME. These authors estimated several parameters of relevance in our calculation, such as additive genetic variance in schizophrenia explained by SNPs at common GWAS arrays equal to 0.3; and additive genetic covariance between schizophrenia and major depression or schizophrenia and bipolar disorder explained by SNPs at common GWAS arrays equal to 0.165 or 0.185, respectively. For power calculation, we assumed an additive genetic covariance between schizophrenia and OCD of 0.15.

## Results

### Genotyping and quality control

We obtained genotypic data for 295 983 SNPs in 433 cases and 484 controls. A total of 144 203 SNPs were monomorphic in our samples. After quality control procedures, the final data set consists of 38 305 SNPs at a frequency higher than 5% in 370 cases and 443 controls. The details on the application of the quality control filters are shown in Supplementary Table 1 and Supplementary Figure 1. Imputation of genotypes reported 368 840 additional SNPs with high imputation quality, MAF>5% and data on more than 95% of the samples. Therefore our final analytic sample contained 407 145 SNPs.

**Figure 1:**
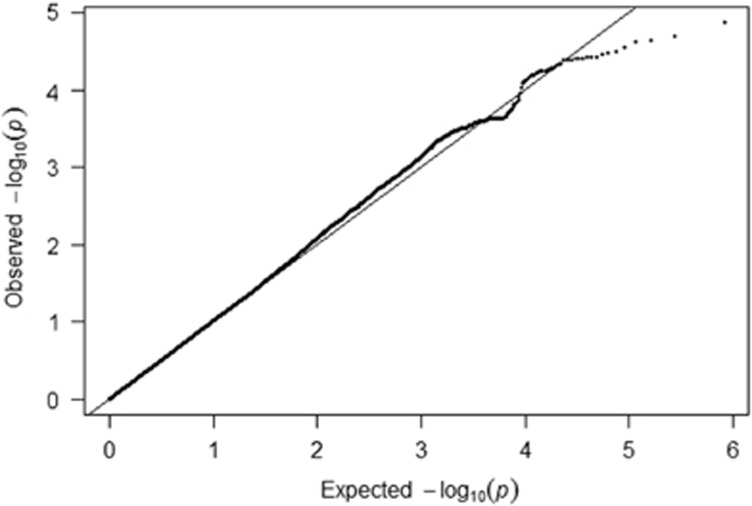
Quantile-quantile plot of the observed versus expected statistic of the OCD study. OCD, obsessive-compulsive disorder.

The population stratification was well controlled with inclusion of the first 10 dimensions of multidimensional scaling, as revealed by a reduction of the stratification factor *λ* from 1.038 to 1.018 (Figure 1).

### Association analyses

**Figure 2:**
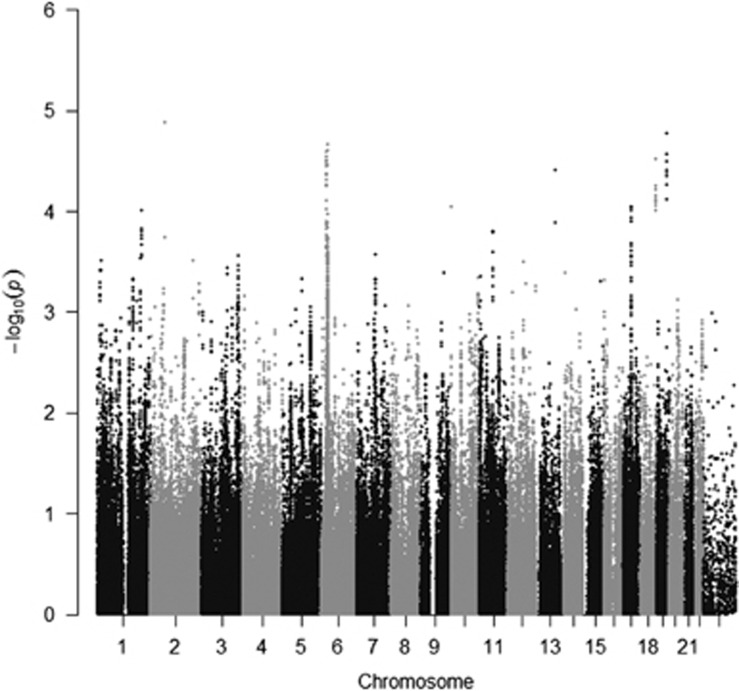
Manhattan plot of genetic associations with OCD showing significance by genomic location. OCD, obsessive-compulsive disorder.

The results for the association test are shown in Figure 2. The most significant SNP (*P*=1.34 × 10^−5^) was rs12151009, a missense variant in *EML2*. Forty-five SNPs at six different regions present *P*<1 × 10^−4^, including two different regions at the major histocompatibility complex (MHC). Table 1 shows association results for the most significant SNP at each region. The regional association plots are shown at Supplementary Figure 2.

The analysis at the gene level, based on 7920 genes with at least two genotyped SNPs, revealed one gene with a *P*-value lower than 1 × 10^−4^, *DNM3* (*P*=7.9 × 10^−5^). Four of the five genotyped SNPs at this gene presented *P*-value for association lower than 0.05, and the linkage disequilibrium among them is very low (Table 2). As a consequence, the gene-based *P*-value was more significant than any of those for individual SNPs.

### Comparison with previous GWAS

We compared our results with the available data from previous GWAS studies: (i) SNPs at *P*<1 × 10^−3^ in meta-analysis of all the samples in the study of Stewart *et al.;*^9^ (ii) SNPs at *P*<1 × 10^−4^ in the study of Matthiesen *et al.*^10^ None of the 33 SNPs reported by Matthiesen *et al.* were presented in our samples. A total of 44 SNPs in our sample are among the 601 reported by Stewart *et al.* Two of them are at *P*<0.05 in our samples, but only one, rs6845865, showed the same direction of association. The C variant is present in 18% of our cases and 14% of our controls. Adding our results to the meta-analysis of Stewart *et al.* increased the significance of the association from 2.5 × 10^−4^ to 5.4 × 10^−5^.The SNP is located within an intron of *ARHGAP10*, 25.3 kb apart from *NR3C2*.

Regarding the gene-based tests, Matthiesen *et al.* reported two experiment-wise significant genes, *C16orf88*, currently known as *KNOP1*, and *IQCK*. The first gene is absent from our data, while *IQCK* lacked any evidence of significance (*P*=0.90).

### Shared polygenic risk between schizophrenia and OCD

**Figure 3:**
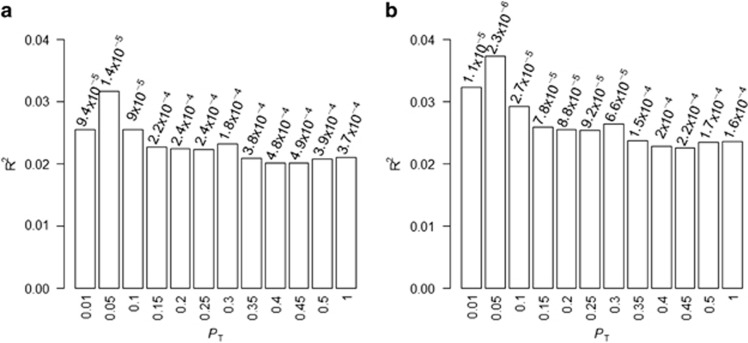
Results of the polygenic risk score analysis using the PGC-SCZ2 data as the discovery sample and OCD data as target sample. The *x* axis represents the different *P*_threshold_. Significance of the score is shown above each column. The *y* axis represents the percentage of variance explained on the observed scale (Nagelkerke's pseudo-*R*^2^). (**a**) Values including the extended MHC region. (**b**) Values after removing the extended MHC region. MHC, major histocompatibility complex; OCD, obsessive-compulsive disorder; PGC-SCZ2, Psychiatric Genetics Consortium schizophrenia data set.

A cross-disorder polygenic risk score analysis, using the schizophrenia data from the PGC-SCZ2 as the discovery sample, and OCD data from our study as the target sample, revealed that the polygenic risk score based on schizophrenia risk is significantly different in OCD subjects than in controls in the expected direction (lowest *P*=1.35 × 10^−5^, reached at *P*_threshold_=0.05, based on 2760 near-independent SNPs), corresponding to 3.17% of variance explained (Figure 3a). Assuming an OR=1 for the first quintile, the fifth quintile presented an OR=2.44 (95% CI=1.52–3.92), Fisher's *P*=1.4 × 10^−4^. None of the 100 random replicates of OCD data, permuting the case–control labels, explained a variance as large as this one (maximum *R*^2^=1.67%, average *R*^2^=0.30%, Supplementary Figure 3A). Removal of the extended MHC region before the analysis improved the significance and percentage of variance explained (lowest *P*=2.3 × 10^−6^ at *P*_threshold_=0.05; *R*^2^=3.7% Figure 3b and Supplementary Figure 3B).

Taking into account the 16 675 nearly independent SNPs in common between PGC-SCZ2 and our OCD data set, and the assumptions described in methodology, the analysis had ~80% power to detect polygenic risk in our OCD samples if the total additive genetic variance explained by SNPs in common is one-fifth of that estimated from common GWAS arrays.

The polygenic risk effect was also detected using the OCD data generated in this work as discovery sample and the schizophrenia data of our previous work as target sample, in spite of the reduced number of SNPs in common for the analysis. The most significant effect (*P*=0.0044 at *P*_threshold_=0.25) explained 1.16% of the variance and is based on 821 near-independent SNPs (Supplementary Figure 4).

## Discussion

In contrast to most of the common psychiatric disorders, where many GWASs have been performed since 2007 including thousands of samples, there are only two published GWASs on OCD, the one by the International OCD Foundation Genetic Collaborative^9^ and the one by the OCD Collaborative Genetics Association Study.^10^ The present work adds new GWAS data to increase our knowledge on the genetics of OCD. Recently, Davis *et al.*^11^ and Yu *et al.*^12^ detected the polygenic risk model in OCD and identified a shared polygenic risk between OCD and Tourette's syndrome. Here, we identified for we believe the first time a shared polygenic risk between OCD and schizophrenia.

The relation between schizophrenia and obsessive-compulsive symptoms is a well-known feature since the beginning of modern psychiatry.^26^ A recent meta-analysis, including 3007 samples from 34 studies, estimated a prevalence of OCD in schizophrenia spectrum disorders of 12.1% (95% CI=7.0–17.1%), although there were a highly significant heterogeneity among studies.^4^ This heterogeneity may be considered an evidence of the difficulties in the study of OCD–schizophrenia co-occurrence. Such difficulties include the fact that obsessions and delusions are not always easy to distinguish, obsessions may appear in response to second-generation antipsychotic treatment, patients with two psychiatric disorders are more likely to seek medical help, there have been changes in hierarchy rules in the different DSM versions, and different assessment instruments may lead to different conclusions.^4, 27^

Recent epidemiological findings using longitudinal nationwide registers from Denmark identified an increase risk of developing schizophrenia in people first diagnosed with OCD (incidence rate ratio=6.90, 95% CI=6.25–7.60), and this risk was approximately twice that of other infancy/adolescent psychiatric disorders such as autism, attention-deficit/hyperactivity disorder or bulimia nervosa.^5^ A parental diagnosis of OCD increased the incidence rate ratio of schizophrenia in the offspring to 4.31 (95% CI=2.72–6.43); and the risk associated to parental diagnosis of OCD was higher than that associated to parental diagnosis of any psychiatric disorder other than schizophrenia or schizophrenia spectrum disorders. Similar results were found in a longitudinal and multigenerational family study using Swedish Patient Register.^6^ Interestingly, OCD-unaffected close relatives of OCD probands had also an increased risk for schizophrenia, and the magnitude of the effect increased as the genetic distance decreased. These facts suggest the existence of shared genetic susceptibility between OCD and schizophrenia spectrum disorders. Our data confirm its existence, providing an explanation for the high co-occurrence of OCD and schizophrenia.

The most strongly associated locus in schizophrenia GWAS is the MHC.^21, 28^ Two of the top results in our GWAS are located in this region. Nevertheless, linkage disequilibrium analysis indicates that our top variants are not related to the main variants involved in schizophrenia susceptibility (Supplementary Figure 5). Furthermore, the polygenic risk is more significant and explains a larger proportion of variance if the MHC region is removed before the analysis. These facts strongly suggest that, although immunity may have a role in both disorders,^29^ this is not due to shared susceptibility variants at the MHC region.

There is a long-lasting debate about the validity of considering a subtype of schizo-obsessive patients in the schizophrenia spectrum.^26, 27, 30, 31^ Schizo-obsessive patients seem to have distinct clinical features, such as higher global, positive and negative symptom severity, more suicide attempts, earlier age at onset or specific cognitive deficits.^26, 32^ According to Poyurovsky *et al.*,^26^ delineation of distinct subgroups of patients on a putative schizophrenia–OCD axis has prognostic and treatment implications, as first-line medications for one disorder can exacerbate the symptoms of the other. Therefore, future improvement of the estimation of the shared polygenic risk, using for instance Bayesian approaches such as the pleiotropy enrichment^33^ or the genomic annotation enrichment,^34^ would be useful in the stratification of OCD patients with psychotic features, or schizophrenic patients with obsessive-compulsive symptoms for more specific treatment.

Interestingly, among the genes at the six different regions at *P*<1 × 10^−4^, there is a gene, *GABBR1*, considered one of the top candidate genes for anxiety disorders based on a convergence functional genomics approach^35^ (Supplementary Figure 2). Comparison with top results in previous GWASs on OCD revealed an interesting SNP near the mineralocorticoid receptor *NR3C2*. This receptor has a role in the hypothalamo–pituitary–adrenal axis response to stress,^36, 37^ a process that may be involved in OCD susceptibility.^38, 39^ The most significant gene in our gene-level analysis was *DNM3*, an interesting gene based on its function as well as pattern of expression. *DNM3* is highly expressed in neurons, where it is involved in clathrin-mediated synaptic vesicle endocytosis.^40^ In addition, a role for *DNM3* in recycling AMPA receptors in dendritic spines has been proposed,^41^ although this result was not confirmed.^40^ New studies are needed to clarify whether *DNM3* has any postsynaptic role.^42^ Several independent SNPs are responsible for the association of *DNM3* in our work, suggesting allelic heterogeneity (Table 2). This is not uncommon in psychiatric genetics. For instance, the existence of different risk alleles at the same locus has been reported previously in the case of schizophrenia at *TCF4* or at the 16p.11.2 locus, among others.^43, 44^ Our result did not reached experiment-wide significance, conservatively set as *P*=6.31 × 10^−6^, requiring further testing in additional data sets.

Taking into account the hypothesis that a subgroup of OCD patients, known as pediatric autoimmune neuropsychiatric disorders associated with streptococcal infections subgroup,^29^ might be related to autoimmunity, the identification of two hits at the MHC in our data may be of relevance. Nevertheless, the absence of similar evidence in previous GWASs of OCD and related disorders, as well as the high density of SNPs at the MHC in the Axiom Exome array, suggest that this may be a chance finding.

The main limit of the study is the reduced sample size, underpowered to identify SNPs associated at genome-wide significant level. In addition, we have used a genotyping array focused on exonic regions instead of genome-wide arrays. Nevertheless, regarding polygenic risk analysis, once a minimum size is achieved for the target sample, the main factor in the detection of polygenic risk is the sample size of the discovery sample.^45^ Our discovery sample was the schizophrenia PGC-SCZ2 data set, that includes 49 case–control sets of samples (comprising 34 241 cases and 45 604 controls) and three family-based sets of samples (1235 parent–affected offspring trios).^24^ Power analysis revealed that our study is well-powered to detect polygenic risk, assuming that additive genetic covariance between schizophrenia and OCD is similar to that between schizophrenia and major depression or bipolar disorder, if the total additive genetic variance explained by SNPs in our data set is at least one-fifth of that estimated from common GWAS arrays. Bearing in mind that there is an important enrichment in GWAS signals around genes,^34^ this assumption seems reasonable. In fact, the intergenic SNPs were depleted of association signals more than 10-fold.^34^ Therefore, polygenic risk analysis may be done on exome arrays, considerably reducing the cost while being able to resolve important questions, such as, in our case, the existence of common SNPs conferring shared risk to schizophrenia and OCD.

In summary, our exome-focused GWASs unveiled *DNM3* as an interesting gene for follow-up studies on the genetic susceptibility to OCD. In addition, the shared polygenic risk between schizophrenia and OCD was detected for the first time. This common genetic susceptibility may be partially responsible for the frequent comorbidity of both the disorders, explaining the epidemiological data on cross-disorder risk.

## Figures and Tables

**Table 1:** Association results for the most significant SNP at those regions with minimal *P*<1 × 10^−4^

*SNP*	*Chr: position (hg19)*	*Alleles*	*MAF*	*OR (95% CI) minor allele*^a^	P-*value*^a^	*Genes in region*
rs12151009	19:46141845	C/T	0.14 (C)	0.50 (0.37–0.68)	1.34 × 10^−5^	*EML2*
rs12327049	18:72260234	C/T	0.20 (C)	0.56 (0.43–0.73)	2.06 × 10^−5^	*CNDP1*, *ZNF407*
rs11685700	2:71162996	A/G	0.44 (A)	1.58 (1.28–1.96)	2.40 × 10^−5^	*VAX2*, *ATP6V1B1*
rs198841	6:26111671	G/T	0.45 (G)	1.56 (1.26–192)	3.27 × 10^−5^	MHC, telomeric edge extended class I subregion
rs9523762	13:93331886	A/G	0.41 (A)	0.66 (0.54–0.81)	6.23 × 10^−5^	*GPC5*
rs114371521	6:29717380	C/T	0.27 (C)	0.59 (0.45–0.76)	7.58 × 10^−5^	MHC, extended class I subregion/classical class I subregion boundary

Abbreviations: Chr, chromosome; CI, confidence interval; MAF, minor allele frequency; MDS, multidimensional scaling; MHC, major histocompatibility complex; OR, odds ratio; SNP, single-nucleotide polymorphism.

a Based on logistic regression using 10 MDS dimensions as covariates.

**Table 2:** Association results for SNPs at *DNM3*

*SNP*	*Position Chr 1 (hg19)*	*Alleles*	*MAF*	*OR (95% CI) minor allele*^a^	P^a^	*SNPs in LD (*r^*2*^)^b^
rs10914144	171.949.750	T/C	0.16 (T)	0.70 (0.53–0.91)	0.0081	
rs17346452	172.053.287	T/C	0.28 (C)	1.10 (0.88–1.37)	0.41	
rs678962	172.189.889	T/G	0.22 (G)	1.41 (1.11–1.79)	0.0047	rs4916245 (0.21)
rs4916245	172.206.026	G/A	0.43 (A)	0.70 (0.57–0.86)	0.00078	rs678962 (0.21)
rs1011731	172.346.548	G/A	0.38 (G)	1.26 (1.03–1.55)	0.026	

Abbreviations: Chr, chromosome; CI, confidence interval; LD, linkage disequilibrium; MAF, minor allele frequency; MDS, multidimensional scaling; OR, odds ratio; SNP, single-nucleotide polymorphism.

a Based on logistic regression using 10 MDS dimensions as covariates.

b *r*^2^ values higher than 0.05 are present.
