# Supplementary figures and images for: Exon-focused genome-wide association study of obsessive-compulsive disorder and shared polygenic risk with schizophrenia
Source: Transl Psychiatry. 2016 Mar 29;6(3):e768–. doi: 10.1038/tp.2016.34 (PMC4872458; doi:10.1038/tp.2016.34)

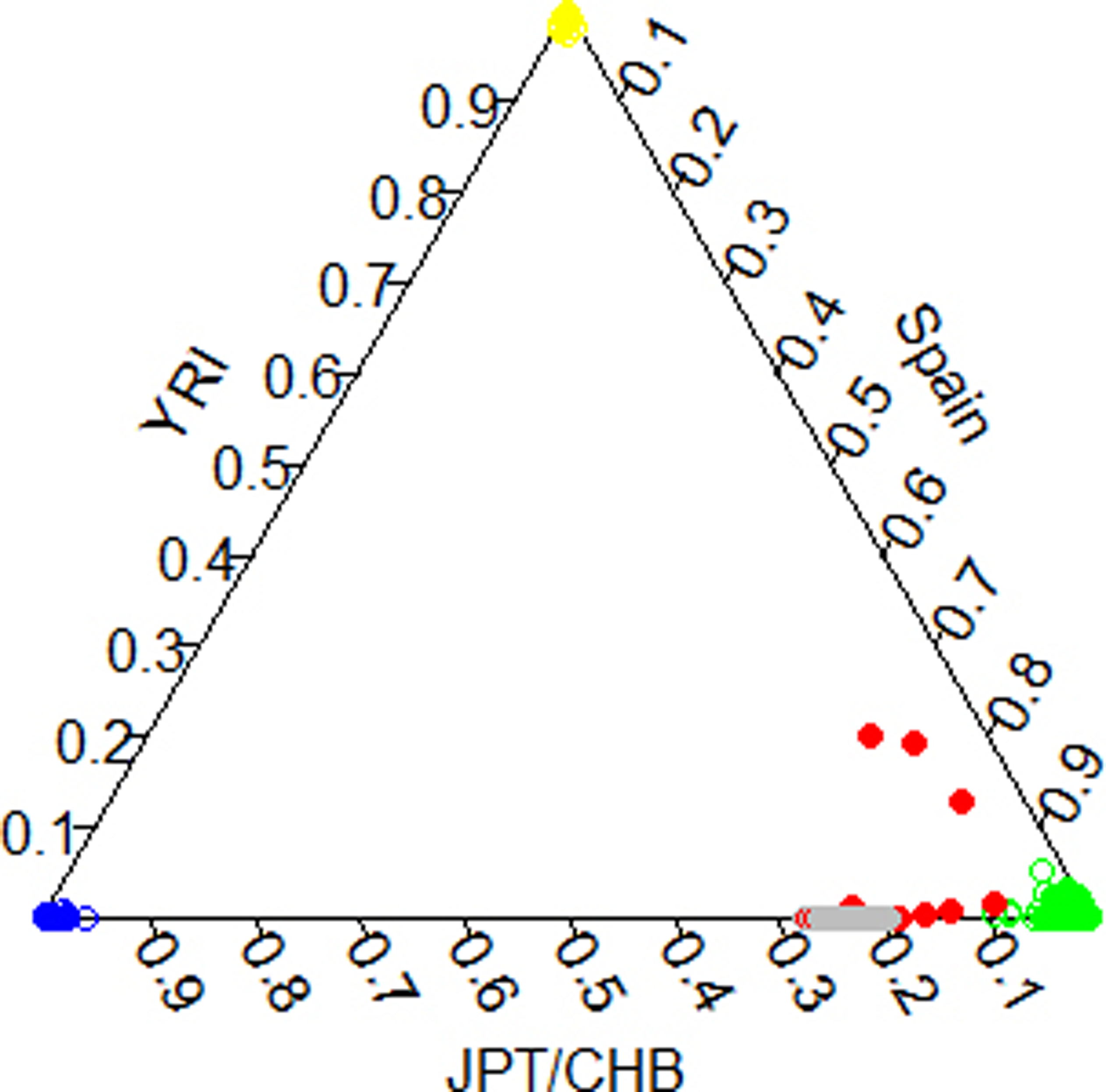

Supplement: Supplementary Figure 1 [file tp201634x1.tif]

Plotted SNPs

|||||

| ||||| ||||

■

||||| | |

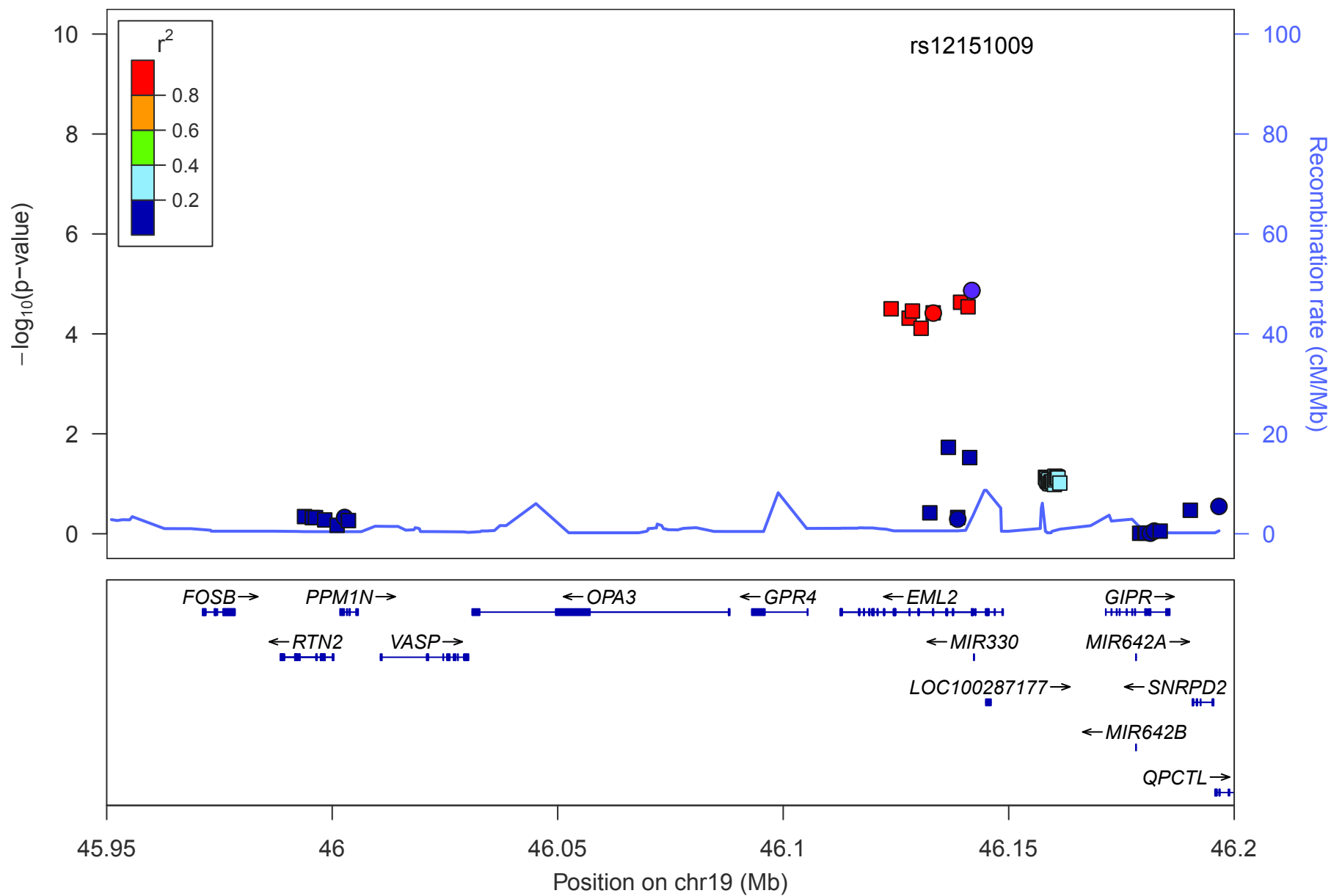

Plotted SNPs

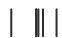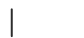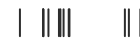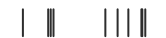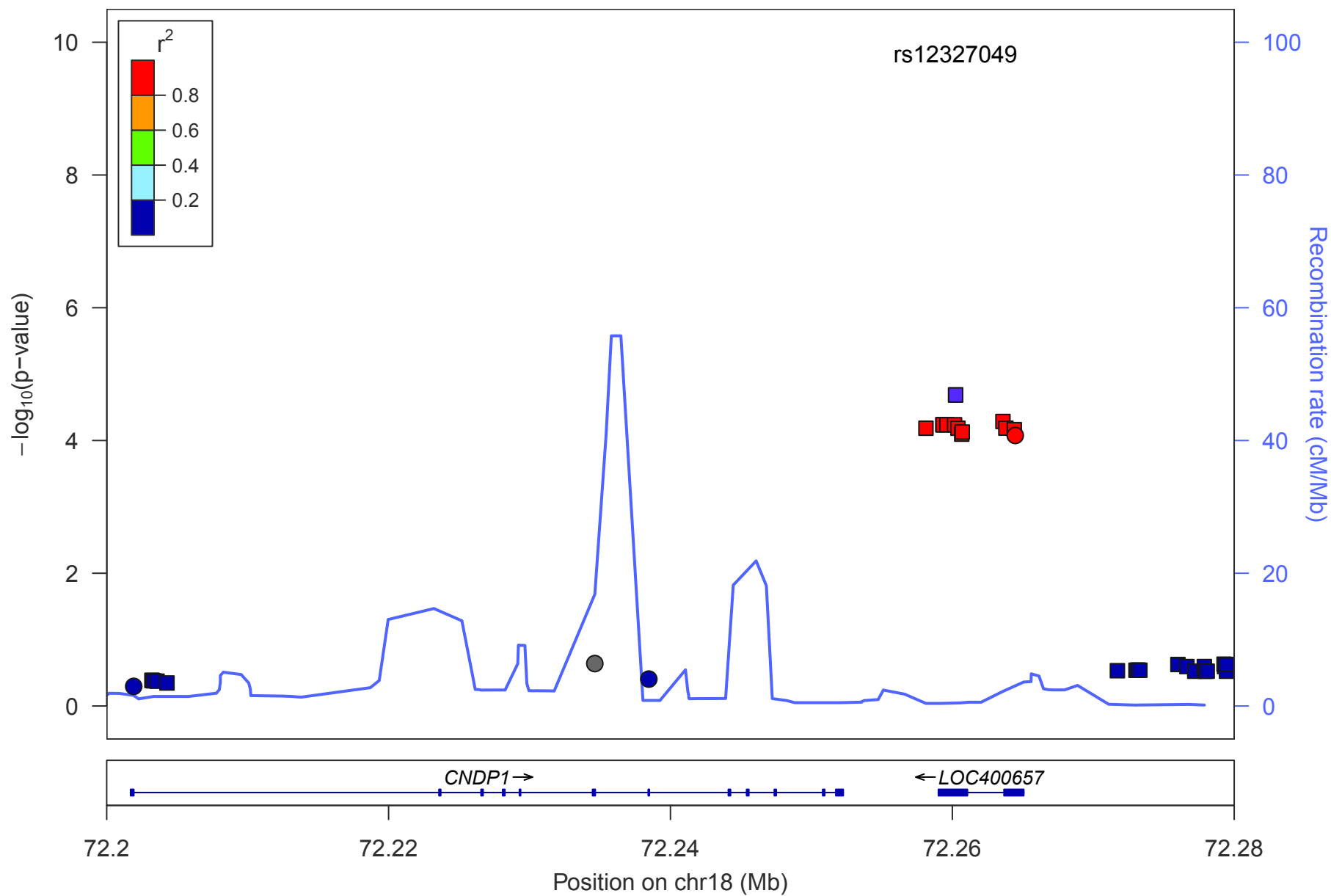

Plotted SNPs

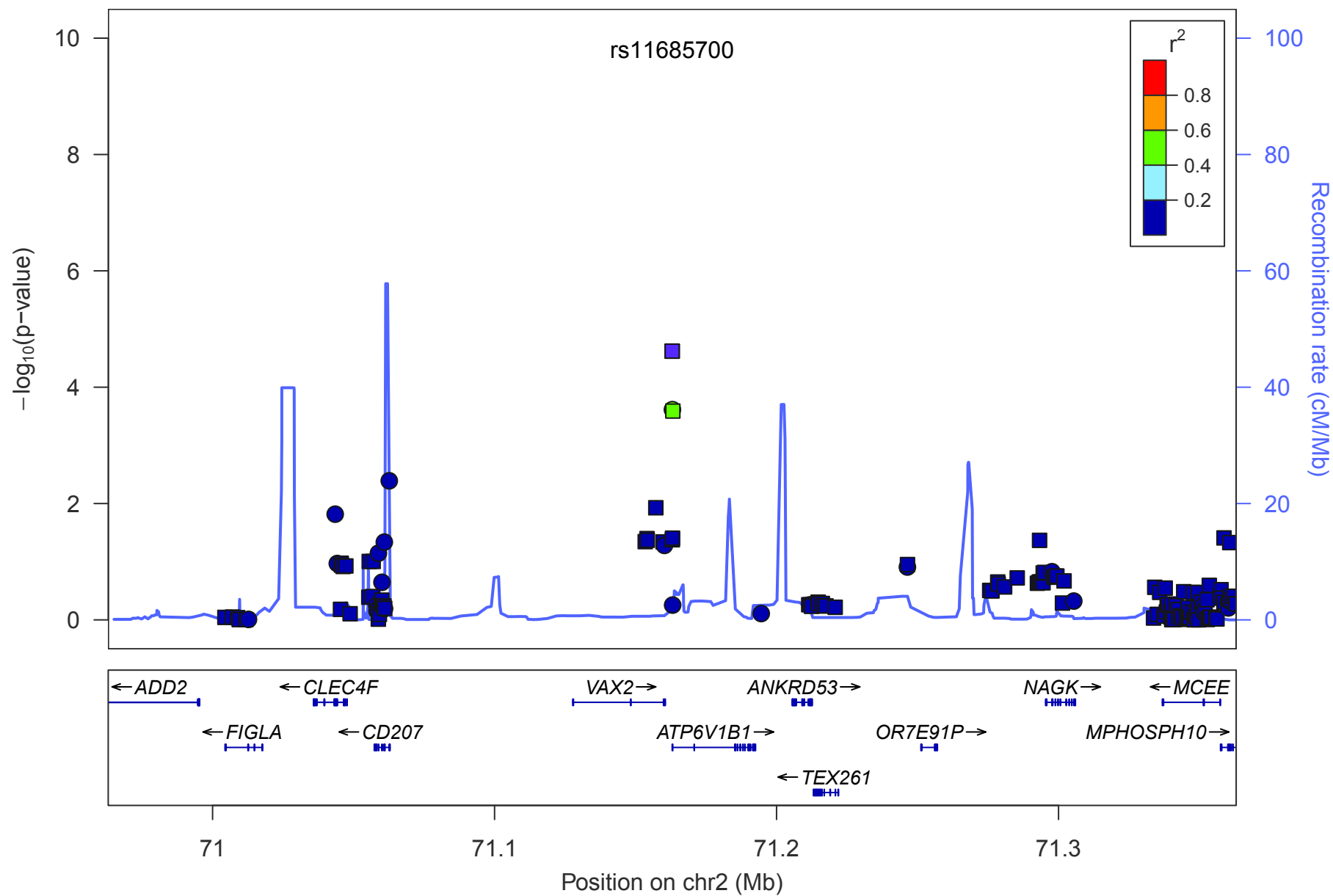

Plotted SNPs | 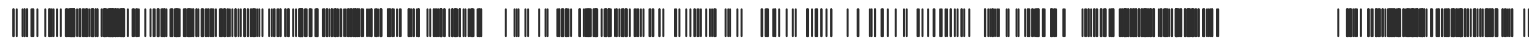

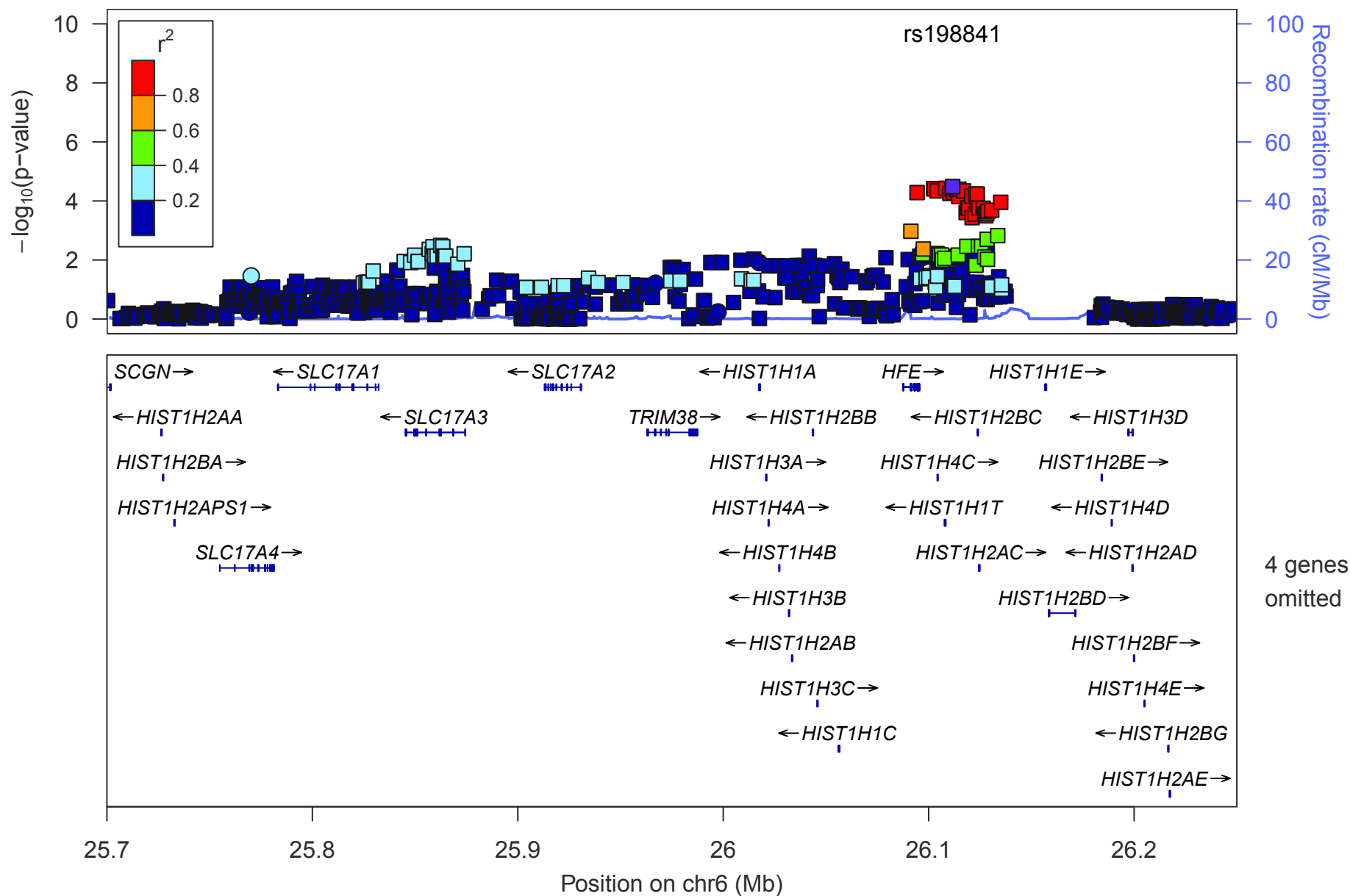

Plotted SNPs

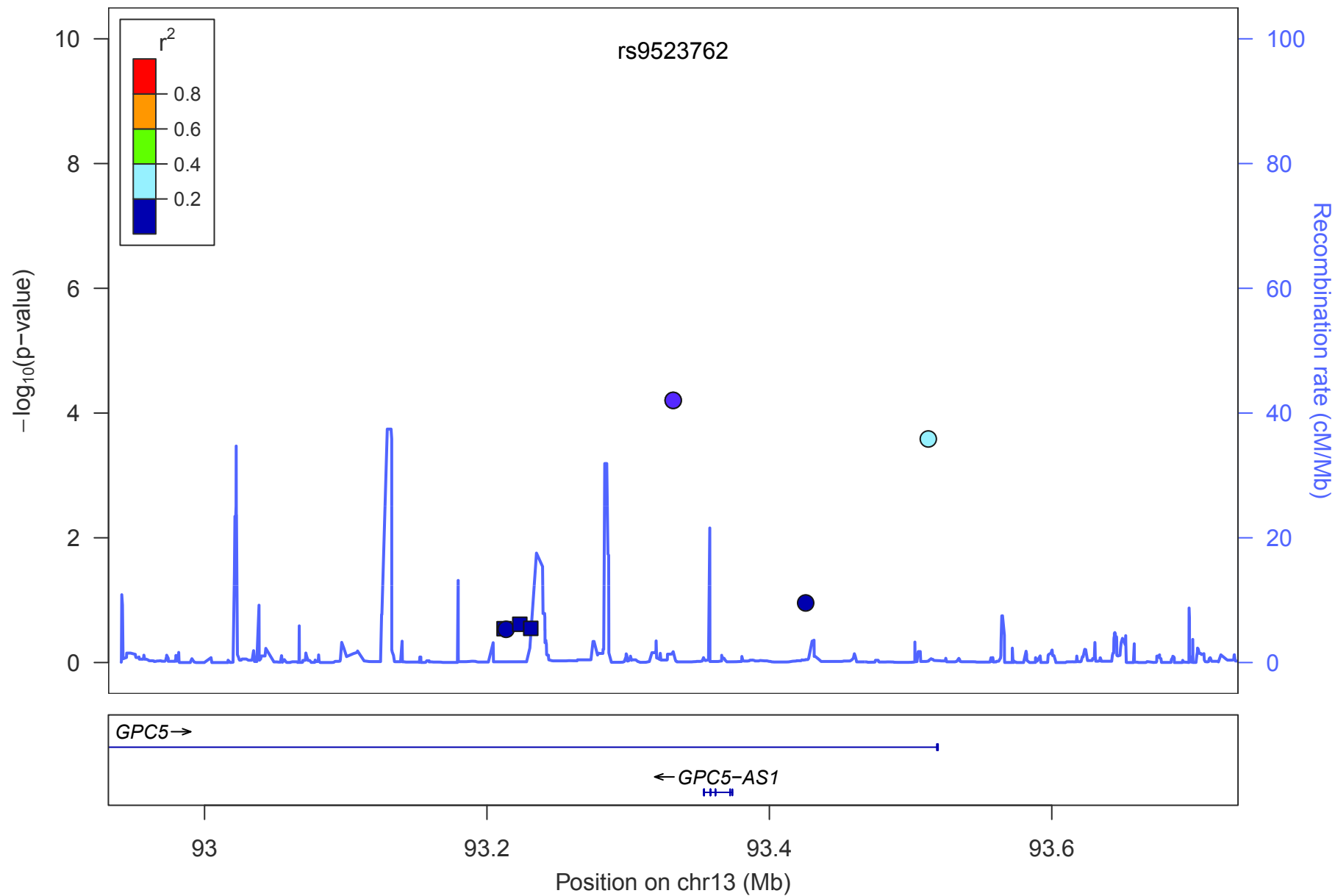

Plotted SNPs

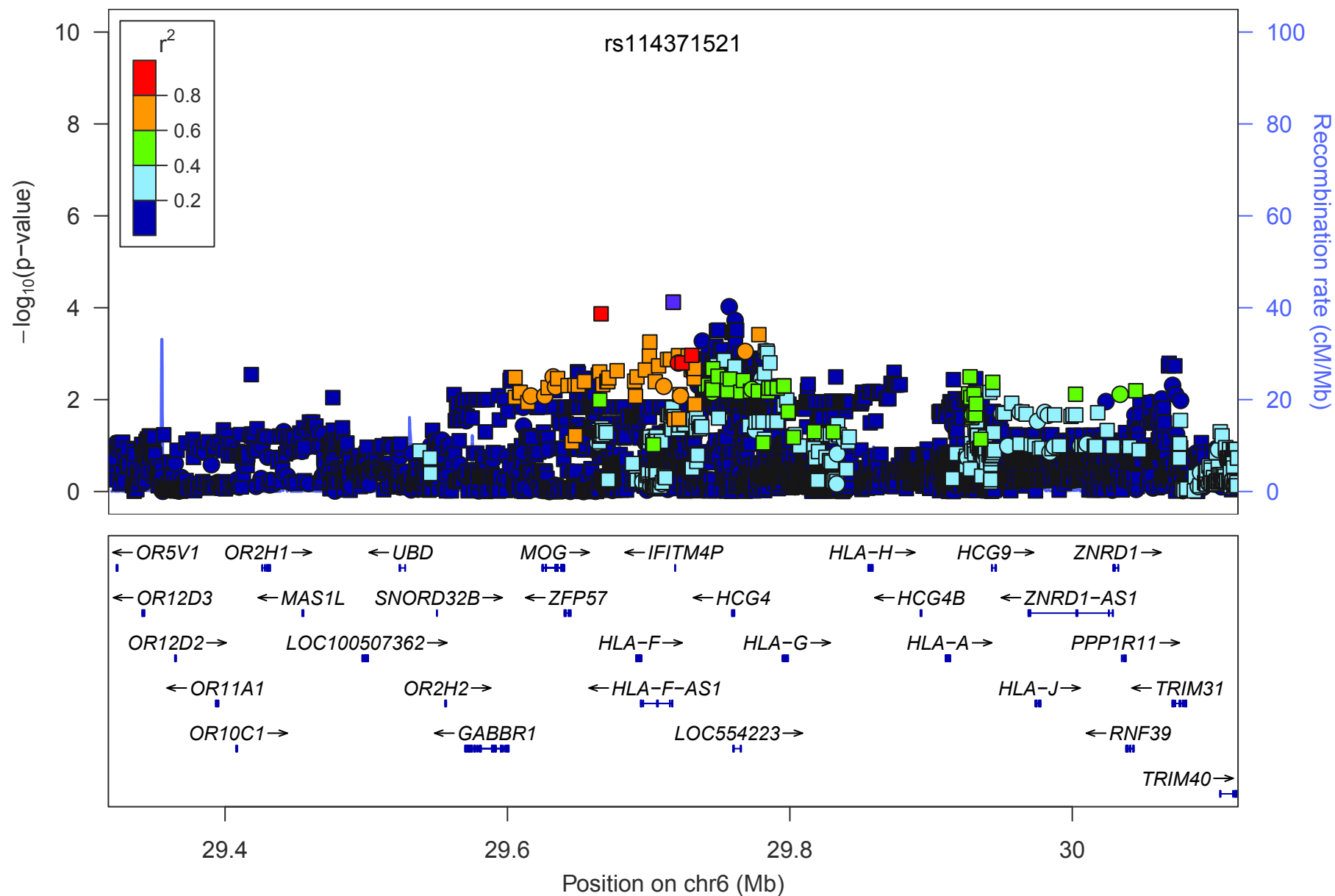

Supplement: Supplementary Figure 2 [file tp201634x2.pdf]

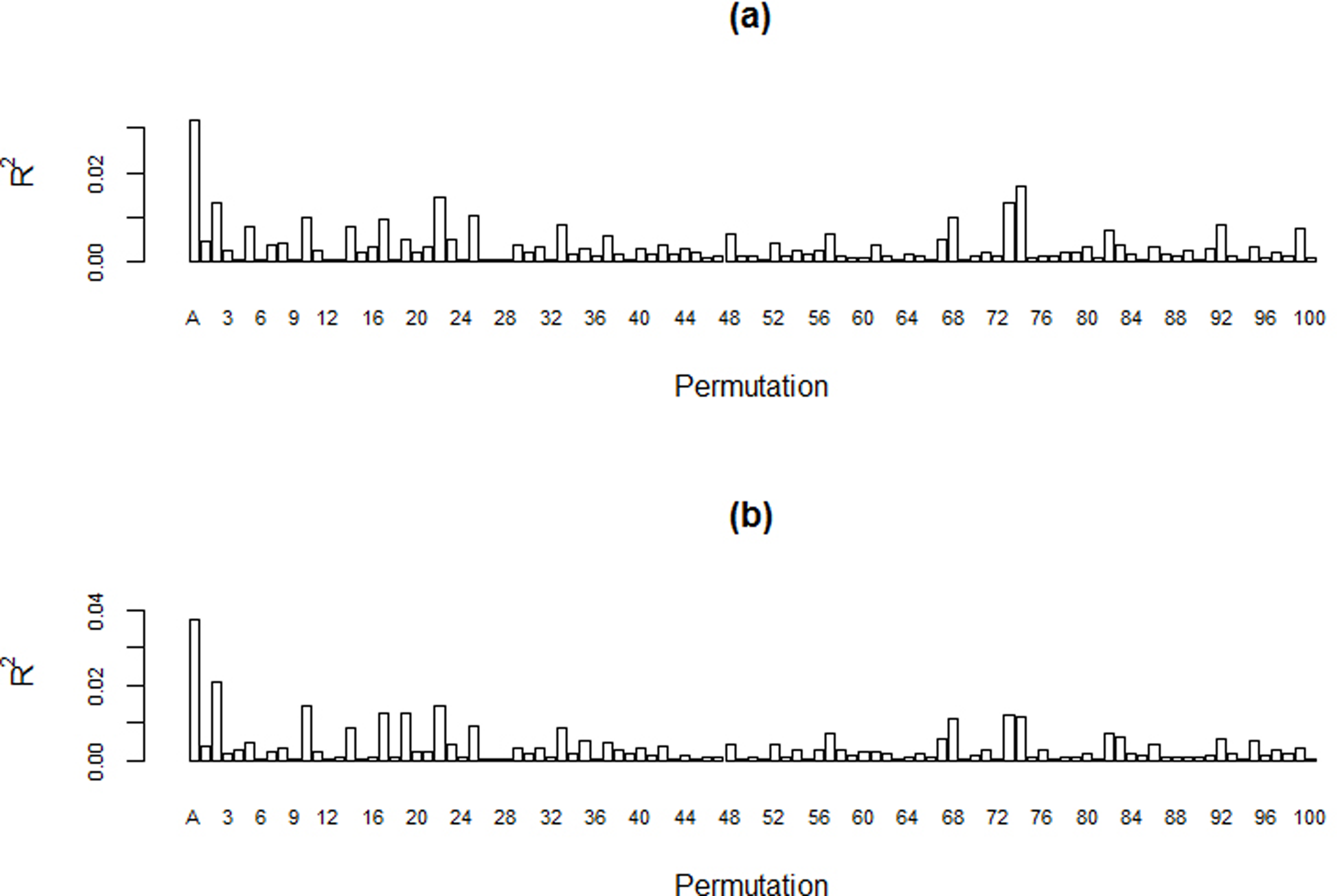

Supplement: Supplementary Figure 3 [file tp201634x3.tif]

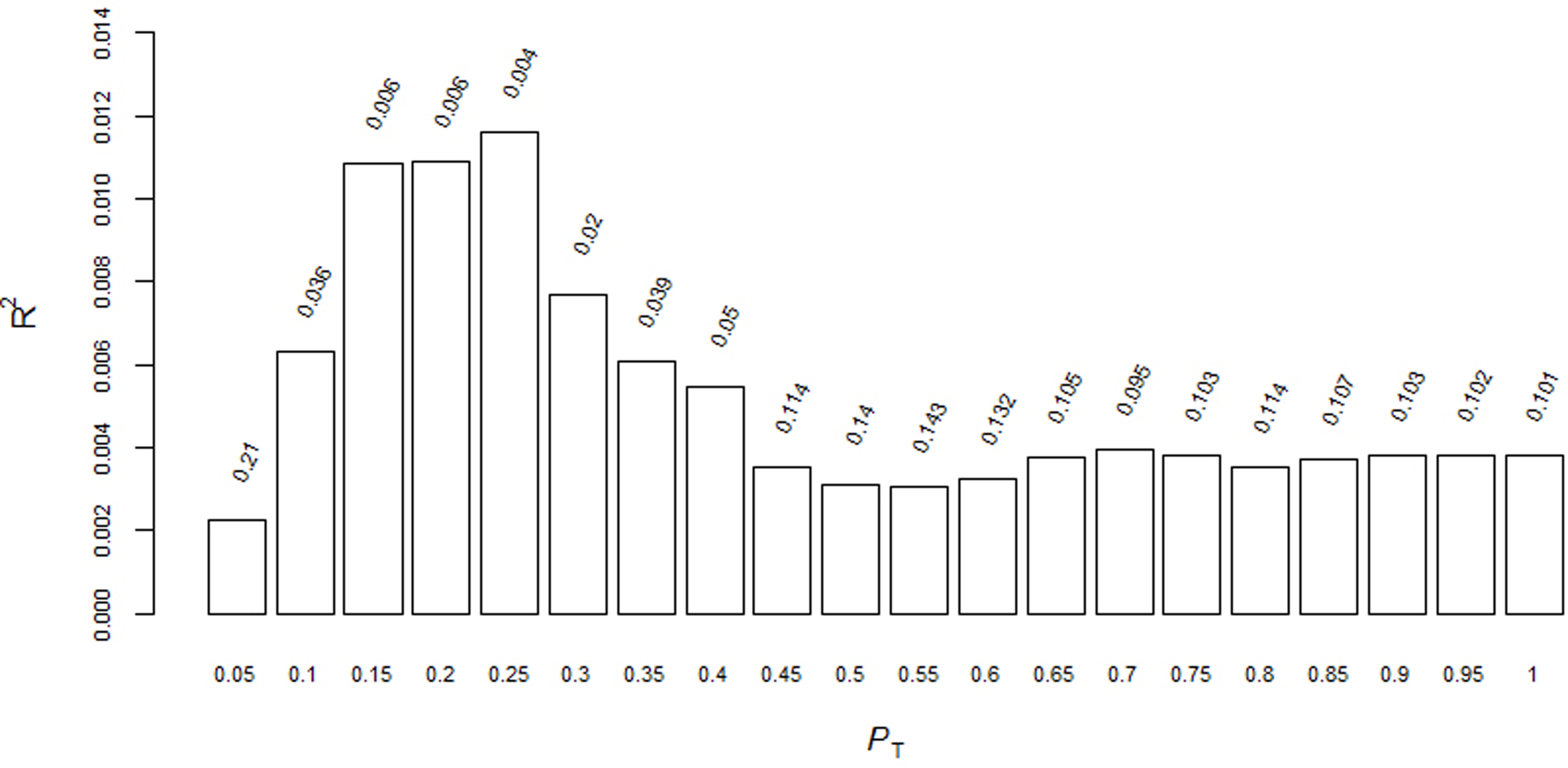

Supplement: Supplementary Figure 1 [file tp201634x4.tif]

(a)

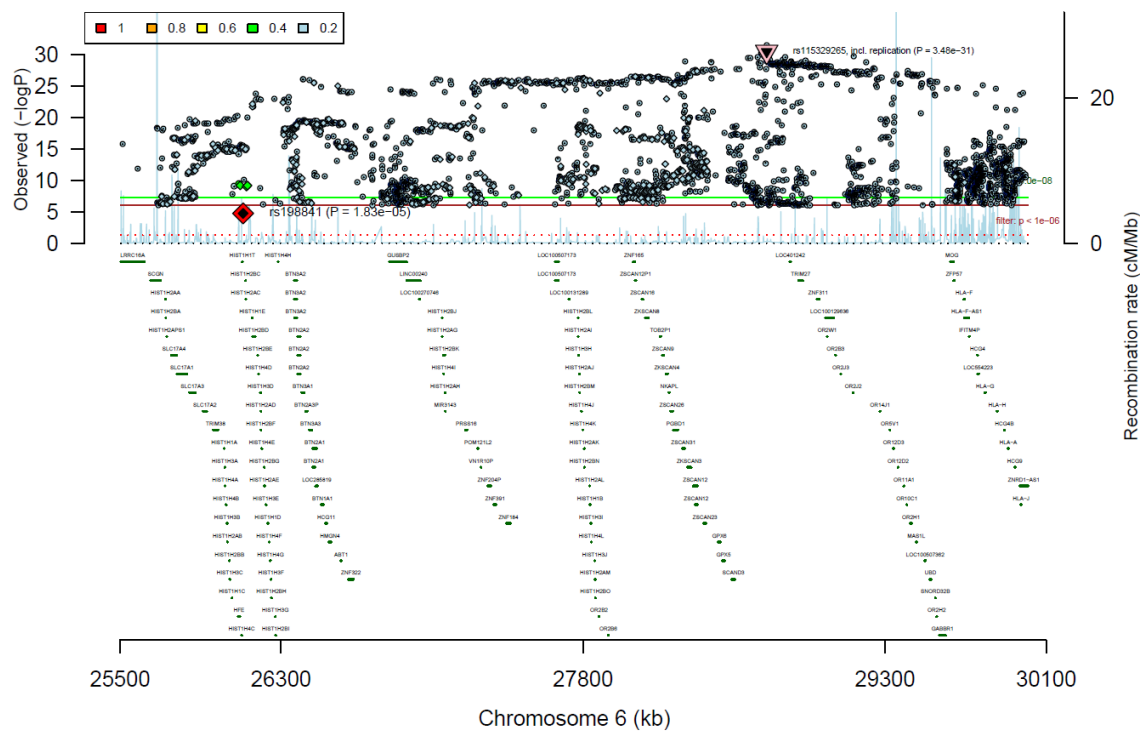

(b)

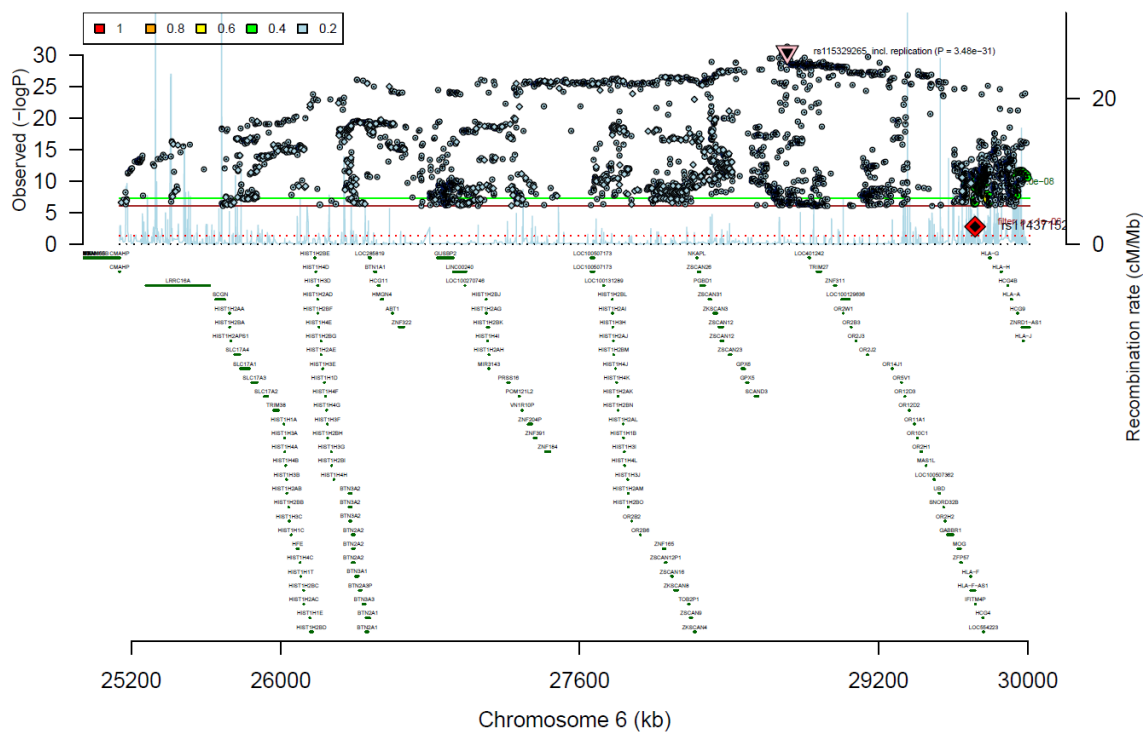

Supplement: Supplementary Figure 2 [file tp201634x5.pdf]
