# Supplementary material for: Exon-focused genome-wide association study of obsessive-compulsive disorder and shared polygenic risk with schizophrenia
Source: Transl Psychiatry. 2016 Mar 29;6(3):e768–. doi: 10.1038/tp.2016.34 (PMC4872458; doi:10.1038/tp.2016.34)
Supplement: Supplementary Table 1 [file tp201634x6.docx]

**Supplementary Table 1.** Number of SNPs and samples after application of quality control filters

| Filter^1^ | SNPs | Controls | Cases |
| --- | --- | --- | --- |
|  | 295 983 | 484 | 433 |
| SNP genotyping call rate < 95% | 290 105 |  |  |
| Hardy-Weinberg equilibrium (P < 0.001) | 289 564 |  |  |
| Different call rate cases vs controls (P < 0.001) | 289 339 |  |  |
| MAF < 5% | 38 305 |  |  |
| Sample genotyping call rate < 95% |  | 477 | 423 |
| Discordant gender |  | 456 | 412 |
| Heterozygosity levels |  | 450 | 395 |
| Cryptic relatedness |  | 445 | 377 |
| Ancestry |  | 443 | 370 |

^1^See the Method section for description of the filters.
