# Supplementary material for: Exon-focused genome-wide association study of obsessive-compulsive disorder and shared polygenic risk with schizophrenia
Source: Transl Psychiatry. 2016 Mar 29;6(3):e768–. doi: 10.1038/tp.2016.34 (PMC4872458; doi:10.1038/tp.2016.34)
Supplement: Supplementary Information [file tp201634x7.docx]

**Supplementary Figure 1.** Results of the ancestry analysis using Structure. Each axis represents the probability of belonging to an specific group. Samples are coded as follows: green, Spanish samples; grey, CEU samples, blue, Asian (JPT/CHB) samples; yellow, YRI samples; red, Spanish samples with less than 90% ancestry in the Spanish cluster. The red samples were removed from the analysis.

**Supplementary Figure 2.** Regional association plots for OCD at the most significant SNPs (*P* < 1x10^-4^). LD values are taken from The 1000 Genomes Project European samples (CEU) and correspond to the value of pairwise *r^2^* between the most significant SNP, shown in purple, and flanking SNPs. The blue line represents recombination rate. Genotyped SNPs are shown as dots and imputed SNPs as squares. Plots were drawn using LocusZoom ( http://locuszoom.sph.umich.edu/locuszoom/) and human genome assembly hg19.

**Supplementary Figure 3.** Results of the polygenic risk score analysis using the PGC-SCZ2 data as discovery and 100 random permutations of the OCD dataset as target, including (A) or excluding (B) the extended MHC region. The x-axis represents the different permutated replicates and the actual value at the first column, labeled as "A". The y-axis represents the percentage of variance explained on the observed scale (Nagelkerke's pseudo-R^2^).

**Supplementary Figure 4.** Results of the polygenic risk score analysis using the OCD data as the discovery sample and our schizophrenia data as target sample. The x-axis represents the different *P_threshold_*. Significance of the score is shown above each column. The y-axis represents the percentage of variance explained on the observed scale (Nagelkerke's pseudo-R^2^).

**Supplementary Figure 5.** Regional association plot of the schizophrenia GWAS from the PGC-SCZ2 along the MHC region (human genome assembly hg18). The plots show the LD related to SNPs rs198841 (A) or rs114371521 (B), the two more significant SNPs at this region in our work. Direction of effect is the opposite to ours for rs198841 and the same for rs114371521. Plots were drawn using Ricopili (http://www.broadinstitute.org/mpg/ricopili/). The most significant SNP of the PGC-SCZ2 mega-GWAS is represent by a triangle.
